# Differential Receptor Binding and Regulatory Mechanisms for the Lymphangiogenic Growth Factors Vascular Endothelial Growth Factor (VEGF)-C and -D*[Fn FN2]

**DOI:** 10.1074/jbc.M116.736801

**Published:** 2016-11-16

**Authors:** Natalia Davydova, Nicole C. Harris, Sally Roufail, Sophie Paquet-Fifield, Musarat Ishaq, Victor A. Streltsov, Steven P. Williams, Tara Karnezis, Steven A. Stacker, Marc G. Achen

**Affiliations:** From the ‡ Tumour Angiogenesis and Microenvironment Program, Peter MacCallum Cancer Centre, Melbourne, Victoria 3000,; the § Florey Institute of Neuroscience and Mental Health, 30 Royal Parade, Parkville, Victoria 3052, and; the ¶ Sir Peter MacCallum Department of Oncology, University of Melbourne, Victoria 3010, Australia

**Keywords:** angiogenesis, endothelial cell, lymphangiogenesis, mutagenesis in vitro, receptor, vascular endothelial growth factor (VEGF)

## Abstract

VEGF-C and VEGF-D are secreted glycoproteins that induce angiogenesis and lymphangiogenesis in cancer, thereby promoting tumor growth and spread. They exhibit structural homology and activate VEGFR-2 and VEGFR-3, receptors on endothelial cells that signal for growth of blood vessels and lymphatics. VEGF-C and VEGF-D were thought to exhibit similar bioactivities, yet recent studies indicated distinct signaling mechanisms (*e.g.* tumor-derived VEGF-C promoted expression of the prostaglandin biosynthetic enzyme COX-2 in lymphatics, a response thought to facilitate metastasis via the lymphatic vasculature, whereas VEGF-D did not). Here we explore the basis of the distinct bioactivities of VEGF-D using a neutralizing antibody, peptide mapping, and mutagenesis to demonstrate that the N-terminal α-helix of mature VEGF-D (Phe^93^–Arg^108^) is critical for binding VEGFR-2 and VEGFR-3. Importantly, the N-terminal part of this α-helix, from Phe^93^ to Thr^98^, is required for binding VEGFR-3 but not VEGFR-2. Surprisingly, the corresponding part of the α-helix in mature VEGF-C did not influence binding to either VEGFR-2 or VEGFR-3, indicating distinct determinants of receptor binding by these growth factors. A variant of mature VEGF-D harboring a mutation in the N-terminal α-helix, D103A, exhibited enhanced potency for activating VEGFR-3, was able to promote increased COX-2 mRNA levels in lymphatic endothelial cells, and had enhanced capacity to induce lymphatic sprouting *in vivo*. This mutant may be useful for developing protein-based therapeutics to drive lymphangiogenesis in clinical settings, such as lymphedema. Our studies shed light on the VEGF-D structure/function relationship and provide a basis for understanding functional differences compared with VEGF-C.

## Introduction

VEGF-C and VEGF-D are secreted protein growth factors that induce proliferation and sprouting of endothelial cells lining blood vessels and lymphatic vessels and promote angiogenesis and lymphangiogenesis in developing tissues and pathologies, such as cancer (1–4). They induce metastasis in animal models of cancer, exhibit expression patterns in a range of human cancers that correlate with parameters of tumor development, and are considered potential targets for therapeutics designed to restrict tumor growth and spread (5–17). VEGF-C and VEGF-D may also play roles in suppressing the immune response to cancer. For example, tumor-derived VEGF-C and associated lymph node lymphangiogenesis suppressed anti-tumor immunity in a murine melanoma model; this type of immunomodulatory effect, involving an immunosuppressive function of lymphatic endothelial cells (LECs),^3^ may be relevant for the design of future immunotherapeutic strategies for cancer (18, 19). In other disease settings, VEGF-C and VEGF-D are being explored in approaches to drive therapeutic angiogenesis and/or lymphangiogenesis for cardiovascular medicine and lymphedema (9, 20, 21).

Both VEGF-C and VEGF-D are initially produced as precursor proteins comprising N- and C-terminal propeptides flanking a central VEGF homology domain (VHD) containing binding sites for VEGFR-2 and VEGFR-3, cell surface receptors on endothelial cells that signal for angiogenesis and lymphangiogenesis (22–24). Proteolytic processing can remove the propeptides to generate mature VEGF-C and VEGF-D, which activate VEGFR-2 and VEGFR-3, thus driving the growth of blood vessels and lymphatics (25–31).

Broadly, VEGF-C and VEGF-D are thought to exhibit similar receptor-binding specificities and biological activities. However, recent findings suggested that there may be differences in the structure/function relationships for these two growth factors. For example, it has been reported that the choice of the N-terminal processing site for the production of mature VEGF-D can profoundly influence receptor specificity (32), whereas there has been no such report for VEGF-C. Further, studies in mouse models of cancer showed that VEGF-C produced by tumor cells promoted expression of COX-2 (an enzyme involved in the biosynthesis of prostaglandins) in the endothelial cells of collecting lymphatic vessels, whereas VEGF-D did not, indicating that these growth factors may exhibit distinct regulatory mechanisms for promoting metastasis via the lymphatic vasculature (5). Differences in the functions of VEGF-C and VEGF-D are important from the perspective of cancer biology, given that these growth factors can exhibit distinct patterns of expression in human tumors (8, 33). For example, VEGF-C has been reported to be up-regulated in head and neck cancer *versus* normal epithelium, whereas VEGF-D expression is down-regulated (34); conversely, VEGF-D, but not VEGF-C, was reported to be an independent predictor of poor outcome in epithelial ovarian carcinoma (35).

The crystal structures of mature human VEGF-C bound to portions of VEGFR-2 and VEGFR-3 have been reported (36, 37), and the crystal structure of a variant of mature human VEGF-D (VEGF-D C117A) has been determined (32). However, there have been no reports of structures for VEGF-D in complex with either VEGFR-2 or VEGFR-3, so the structural determinants important for the interaction of VEGF-D with its receptors remain to be fully characterized. Here we identify amino acid residues in the N-terminal α-helix of mature VEGF-D that are critical for receptor binding and the bioactivities of this protein. We show that the comparable region of VEGF-C is not a key determinant of receptor binding, which indicates divergent mechanisms for receptor interactions in VEGF-C *versus* VEGF-D. Our findings have potential clinical significance for developing monoclonal antibodies to block VEGF-D in cancer and for optimizing protein growth factors to promote therapeutic lymphangiogenesis and lymphatic remodeling to treat lymphedema and inflammatory conditions.

## Results

### 

#### 

##### Mapping the Binding Site in VEGF-D of an Antibody That Blocks Interactions with VEGFR-2 and VEGFR-3

**FIGURE 1.**
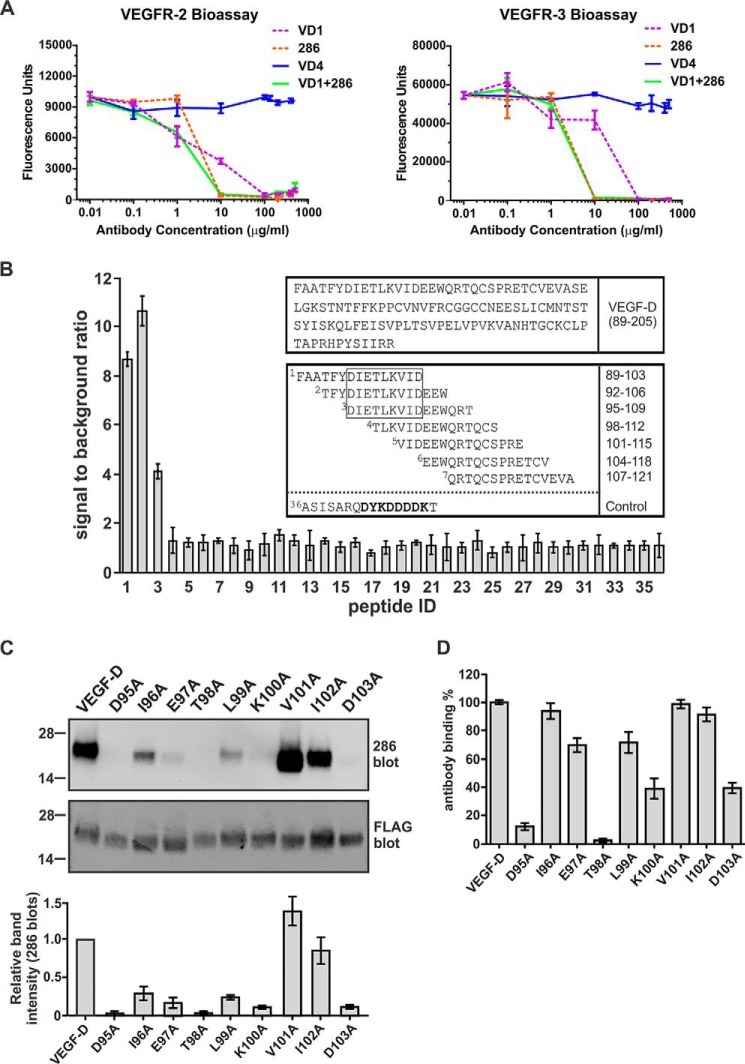
**Neutralizing effect of mAb 286, mapping of its binding site, and analysis of binding to VEGF-D variants with mutated residues in N-terminal α-helix.** *A*, the capacity of mAb 286 to block binding and cross-linking, by VEGF-DΔNΔC, of chimeric receptors containing VEGFR-2 (*left*) or VEGFR-3 (*right*) extracellular domains was assessed in bioassays (see “Experimental Procedures”). Also included were neutralizing mAb VD1, which binds loop 2 of VEGF-DΔNΔC, and mAb VD4, which binds, but does not neutralize, VEGF-DΔNΔC (39). *B*, peptide-based mapping of the mAb 286 binding site in VEGF-DΔNΔC by ELISA (see “Experimental Procedures”). The ratio of signal to background for the interaction of mAb 286 with immobilized peptides is shown on the *y* axis of the graph, and the *x* axis indicates the identifier numbers of peptides. *Top box above* the *graph*, amino acid sequence for the VEGF homology domain of human VEGF-D; N-terminal residue (phenylalanine) is number 89, and the C-terminal residue (arginine) is 205. *Bottom box above* the *graph*, examples of peptides used in mapping (mAb 286 binding site is in a *rectangle*). The FLAG sequence is shown in *boldface type* in peptide 36, which lacks the VEGF-D-derived sequence, and was the negative control. *C*, detection of VEGF-DΔNΔC variants by Western blotting under reducing and denaturing conditions using mAb 286 (*top*) or M2 anti-FLAG mAb as a positive control (*bottom*). Each well contained 30 ng of purified protein. *VEGF-D*, VEGF-DΔNΔC; variants of this protein each have one residue mutated to alanine, as indicated. Positions of molecular mass markers (in kDa) are shown to the *left*. The *histogram under* the *blots* shows intensities of bands for VEGF-D variants (mean ± S.D.) relative to the intensity of the band for VEGF-DΔNΔC, as determined from Western blots with mAb 286. *D*, analysis of mAb 286 binding to VEGF-DΔNΔC variants by ELISA. M2 was used for capture and mAb 286 for detection; the *y* axis shows binding of variant proteins compared with VEGF-DΔNΔC (the latter defined as 100% binding), and the *x* axis lists VEGF-D variants. Equal amounts of VEGF-DΔNΔC and variants were used. For *A*, *B*, and *D*, assays were conducted three times. *Columns*, mean; *error bars*, S.D.

We previously employed a neutralizing monoclonal antibody (mAb) to mature human VEGF-D, designated VD1, to identify part of the binding site in VEGF-D for VEGFR-2 and VEGFR-3. The region thus identified, ^147^NEESL^151^, was located in the L2 loop on the pole of the VEGF-D monomer (38). To identify other regions of VEGF-D critical for receptor interactions and the distinct biological activities of this growth factor, we assessed a panel of commercially available and in-house VEGF-D mAbs for neutralizing capacity in bioassays of binding and cross-linking of VEGFR-2 and VEGFR-3. These assays employed cell lines expressing chimeric receptors consisting of the entire extracellular domain of VEGFR-2 or VEGFR-3 and the trans-membrane and cytoplasmic domains of the mouse erythropoietin receptor (25). Binding and cross-linking of the chimeric receptors allows these cells to survive and proliferate in the absence of interleukin-3 (IL-3). This analysis demonstrated that the commercially available mAb 286 blocks binding and cross-linking of both VEGFR-2 and VEGFR-3 by a form of mature human VEGF-D previously designated VEGF-DΔNΔC (22) (Fig. 1*A*). The neutralizing VD1 mAb was included as a positive control, which blocked binding and cross-linking of both receptors by VEGF-DΔNΔC, as reported previously (39). The VD4 mAb, which binds VEGF-DΔNΔC but does not block the interactions of this ligand with VEGFR-2 or VEGFR-3, was also included and had no effect on receptor binding and cross-linking in the bioassays, as expected (39).

We mapped the binding site of mAb 286 by ELISA using a synthetic peptide library covering the amino acid sequence of VEGF-DΔNΔC (Fig. 1*B*). Positive signals were detected for interactions of mAb 286 with three peptides that had the sequence ^95^DIETLKVID^103^ in common. This sequence is located near the N terminus of VEGF-DΔNΔC and lies in the N-terminal α-helix of mature VEGF-D (32). To confirm these findings, we generated mutants of VEGF-DΔNΔC with each residue in the ^95^DIETLKVID^103^ region individually converted to alanine. The interaction of these mutants with mAb 286 was monitored by Western blotting and ELISA, which demonstrated that various residues in this region are important for binding this mAb. For example, mutation of either Asp^95^ or Thr^98^ to alanine completely abrogated binding to mAb 286, as assessed by Western blotting (Fig. 1*C*), and almost completely abrogated binding in the ELISA (Fig. 1*D*). Mutation of Glu^97^, Leu^99^, Lys^100^, or Asp^103^ to alanine reduced binding to mAb 286, as assessed by both methods, but not to the same degree as Asp^95^ or Thr^98^. These findings confirm the importance of the ^95^DIETLKVID^103^ region in the N-terminal α-helix of mature VEGF-D for the interaction with mAb 286. Our results also show that targeting this region of mature VEGF-D with a mAb can prevent this growth factor from binding and cross-linking VEGFR-2 and VEGFR-3.

##### Identification of Residues in the N-terminal α-Helix Critical for Receptor Activation

**FIGURE 2.**
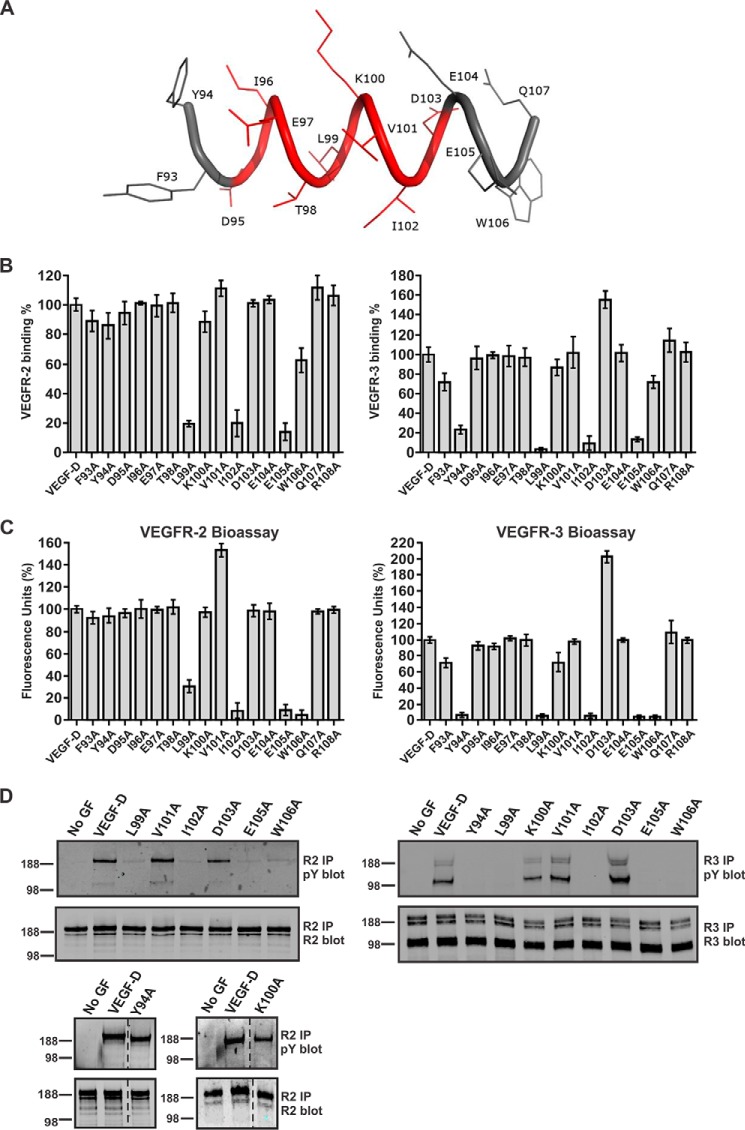
**Interaction of VEGFR-2 and VEGFR-3 with VEGF-DΔNΔC variants.** *A*, representation of structure for part of the N-terminal α-helix (^93^FYDIETLKVIDEEWQ^107^) in human mature VEGF-D with the mAb 286 binding site shown in *red. B*, analysis of binding of VEGF-DΔNΔC variants to VEGFR-2 (*left*) and VEGFR-3 (*right*) by ELISA (see “Experimental Procedures”). *y* axes show binding of variant proteins compared with VEGF-DΔNΔC (the latter defined as 100%), and *x* axes define the mutated amino acid in each variant. The same amount of each VEGF-DΔNΔC variant was used. *VEGF-D*, VEGF-DΔNΔC. Assays were conducted three times. *Columns*, mean; *error bars*, S.D. *C*, bioassays for binding and cross-linking of the extracellular domains of VEGFR-2 (*left*) and VEGFR-3 (*right*) by VEGF-DΔNΔC variants. The same amount of each VEGF-DΔNΔC variant was used in each assay. Results are expressed as a percentage of fluorescence units generated by VEGF-DΔNΔC variants relative to VEGF-DΔNΔC (*y* axes). *x* axes define the mutated amino acid in each variant. Assays were conducted five times. *Columns*, mean; *error bars*, S.D. *D*, receptor phosphorylation induced by selected VEGF-DΔNΔC variants. Adult LECs were stimulated with matched quantities of VEGF-DΔNΔC or its variants or left unstimulated (*No GF*). Lysates were immunoprecipitated (*IP*) with an antibody against VEGFR-2 (*left*) or VEGFR-3 (*right*) and analyzed by reducing SDS-PAGE and Western blotting with an antibody against phosphotyrosine (*pY*) to assess activation of receptors (*top blot* in each pair) or with an antibody against VEGFR-2 (*bottom blot* in each pair on the *left*) or VEGFR-3 (*right bottom blot*) to confirm the presence of each receptor. VEGFR-2 migrated predominantly at ∼230 kDa, whereas VEGFR-3 migrated as three bands, a ∼125-kDa cleaved form and two uncleaved forms of ∼175 and ∼195 kDa that differed in degree of glycosylation. Sizes of molecular mass markers (in kDa) are shown to the *left* of the *panels. Dotted lines* indicate where irrelevant tracks have been excised from images.

The data presented above indicate that mAb 286 blocks the interactions of VEGF-D with VEGFR-2 and VEGFR-3 by binding to the N-terminal α-helix of mature VEGF-D. However, it was not known whether this mAb binds the same or overlapping sites on VEGF-D as these receptors or if binding occurs at distinct sites and the neutralizing effect of the mAb is due to steric hindrance. To explore the importance of specific amino acid residues in the N-terminal α-helix of mature human VEGF-D for receptor binding and activation, we studied VEGF-D mutants in which each residue from position 93 to 108 had been individually altered to alanine (see Fig. 2*A* for locations of these residues). We tested binding of VEGF-DΔNΔC variants to both VEGFR-2 and VEGFR-3 in receptor-binding ELISAs and in bioassays of receptor binding and cross-linking. These data showed that alteration to alanine of each of the residues from Phe^93^ to Thr^98^ (*i.e.* the first six residues of the structure shown in Fig. 2*A*) had no effect on the interaction with VEGFR-2 (Fig. 2, *B* and *C*, *left panels*), whereas for VEGFR-3, alteration of Tyr^94^ to alanine led to a dramatic decrease of receptor binding and cross-linking (Fig. 2, *B* and *C*, *right panels*). Similar loss of VEGFR-3 binding and cross-linking was seen with the L99A mutant, and this mutant also exhibited decreased binding and cross-linking of VEGFR-2. Likewise, alteration of residues C-terminal to Leu^99^ that reduced VEGFR-3 binding and cross-linking (*e.g.* I102A, E105A, and W106A) also reduced binding and cross-linking of VEGFR-2. Interestingly, the D103A mutant exhibited enhanced binding and cross-linking of VEGFR-3, but not VEGFR-2, compared with VEGF-DΔNΔC. We also analyzed the capacity of selected VEGF-D mutants to activate VEGFR-2 and VEGFR-3 on human adult lymphatic endothelial cells (AdLECs) by monitoring tyrosine phosphorylation of these receptors (Fig. 2*D*). The results were consistent with the ELISAs and bioassays (*i.e.* Y94A promoted phosphorylation of VEGFR-2, but not VEGFR-3, whereas L99A, I102A, E105A, and W106A were unable to promote pronounced phosphorylation of either receptor).

**FIGURE 3.**
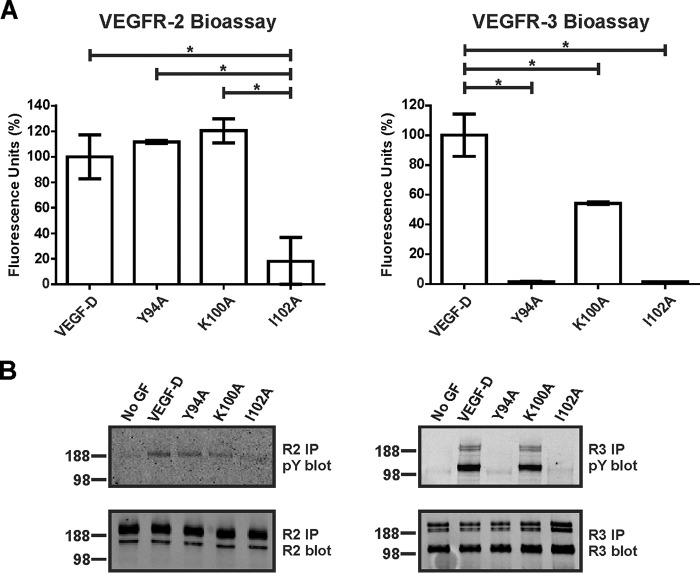
**Receptor binding and activation by untagged VEGF-D variants.** *A*, bioassays for binding and cross-linking of extracellular domains of VEGFR-2 (*left*) and VEGFR-3 (*right*) with altered versions of VEGF-DΔNΔC, Y94A, K100A, and I102A lacking FLAG tag. The same amount of each VEGF-DΔNΔC variant was used. Results are expressed as a percentage of fluorescence units generated relative to untagged VEGF-DΔNΔC (*y* axis). *VEGF-D*, untagged form of VEGF-DΔNΔC. Assays were conducted three times. *Columns*, mean; *error bars*, S.D. *, statistically significant differences as assessed by one-way analysis of variance with Tukey's post hoc test. *B*, adult LECs were stimulated with matched quantities of untagged variants or left unstimulated (*No GF*). Lysates were immunoprecipitated (*IP*) with antibody against VEGFR-2 (*left*) or VEGFR-3 (*right*) and analyzed by reducing SDS-PAGE and Western blotting with antibody against phosphotyrosine (*pY*) to assess receptor activation (*top blots*) or with antibody against VEGFR-2 (*bottom left blot*) or VEGFR-3 (*bottom right blot*) to confirm the presence of each receptor. Sizes of molecular mass markers (in kDa) are shown to the *left* of the *panels*.

The VEGF-DΔNΔC variants studied above had been tagged at the N terminus with the FLAG octapeptide, in relatively close proximity to the N-terminal α-helix, to facilitate purification and quantitation. To confirm that the effects on receptor binding and activation that we observed were not influenced by the FLAG tag, we analyzed the Y94A, K100A, and I102A mutations in the setting of an altered form of VEGF-DΔNΔC that lacked the FLAG tag. Analysis of these mutants in bioassays and receptor phosphorylation assays showed the same profile of receptor binding, cross-linking, and activation as for the corresponding FLAG-tagged mutants (Fig. 3). These findings indicate that the FLAG tag in the VEGF-DΔNΔC variants did not influence the results of our receptor interaction studies.

The data described above demonstrate that residues in the N-terminal α-helix of mature human VEGF-D are critical for binding VEGFR-2 and VEGFR-3 as well as mAb 286. Hence, this mAb interacts with a region of VEGF-D that overlaps part of the binding sites for these receptors. The data also suggest that the N-terminal portion of this α-helix (*i.e.* from Phe^93^ to Thr^98^) is more important for the binding of VEGF-D to VEGFR-3 than to VEGFR-2.

##### Distinct Receptor-binding Determinants in the N-terminal α-Helices of Mature VEGF-D and VEGF-C

**FIGURE 4.**
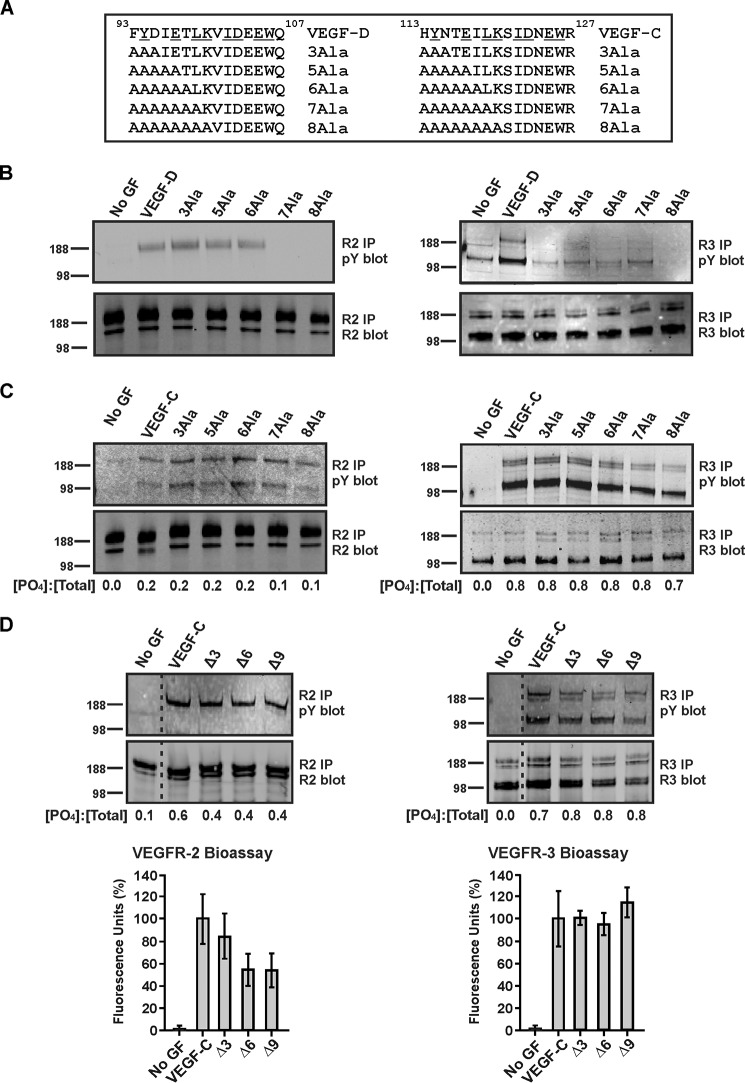
**Effects of mutating residues in N-terminal α-helices of VEGF-DΔNΔC or VEGF-CΔNΔC.** *A*, sequences within the N-terminal α-helices of human VEGF-DΔNΔC (*VEGF-D*) and VEGF-CΔNΔC (*VEGF-C*) (*top*, with identical residues *underlined*) with variants in which multiple residues were altered to alanine shown *below. B* and *C*, blots show analyses of receptor phosphorylation by variants of VEGF-DΔNΔC and VEGF-CΔNΔC, respectively. *D*, blots show analyses of receptor phosphorylation induced by VEGF-CΔNΔC and mutants of VEGF-CΔNΔC lacking residues 113–115 (designated Δ3), 113–118 (Δ6), and 113–121 (Δ9). Graphs *below* blots show the results of bioassays of binding and cross-linking of VEGFR-2 and VEGFR-3 extracellular domains by VEGF-C variants (data are mean percentage of fluorescence relative to VEGF-CΔNΔC ± S.D.). For blots in *B–D*, adult LECs were stimulated with VEGF-DΔNΔC, VEGF-CΔNΔC or their variants or left unstimulated (*No GF*). Lysates were immunoprecipitated with antibody against VEGFR-2 (*left-hand blots*) or VEGFR-3 (*right-hand blots*) and analyzed by reducing SDS-PAGE and Western blotting with antibody against phosphotyrosine to assess receptor activation (*top blots*) or with antibody against VEGFR-2 (*bottom left blots*) or VEGFR-3 (*bottom right blots*) to confirm the presence of each receptor. Sizes of molecular mass markers (in kDa) are shown to the *left* of the blots. The amounts of VEGF-D or VEGF-C variants were matched in each experiment. *Dotted lines* indicate where irrelevant tracks have been excised from the images. In *C* and *D*, *numbers under* the *lanes* of blots represent the ratios of the intensities of phosphorylated receptor signals to intensities of total receptor signals ([PO_4_]/[total]) for each ligand treatment as determined by calculating the mean ratios from two independent experiments. The ratios for VEGFR-2 were derived by combining the intensities of the signals for bands in the size range of 188–230 kDa (note that the lower band of ∼125 kDa in the *top left blot* of *C* was not used because it probably represents co-immunoprecipitated VEGFR-3 arising from receptor heterodimers, as reported previously (68)), whereas those for VEGFR-3 are based on combining the intensities of the ∼125-, ∼175-, and ∼195-kDa forms of this receptor. *pY*, phosphotyrosine; *IP*, immunoprecipitation.

Comparison of the amino acid sequences of the N-terminal α-helices in mature human VEGF-C and VEGF-D indicates a high degree of homology between these regions with multiple residues that are important for the interaction of VEGF-D with VEGFR-2 and/or VEGFR-3 being conserved in VEGF-C (*i.e.* Tyr^94^, Leu^99^, Ile^102^, Glu^105^, and Trp^106^ (Fig. 4*A*). To compare the role of the α-helices in receptor binding, we generated a series of mutants of VEGF-CΔNΔC and VEGF-DΔNΔC in which different parts of these regions were converted to alanine residues (these mutants are defined in Fig. 4*A*). These mutants were tested for their ability to activate VEGFR-2 and VEGFR-3 by monitoring tyrosine phosphorylation of these receptors.

Mutation to alanine of all six residues N-terminal to Leu^99^ (designated 6Ala) in VEGF-DΔNΔC did not alter the capacity to promote tyrosine phosphorylation of VEGFR-2 (Fig. 4*B*, *left*). However, additional alteration of either Leu^99^ (7Ala) or both Leu^99^ and Lys^100^ (8Ala) to alanine prevented VEGFR-2 phosphorylation. Notably, exchange of only Phe^93^, Tyr^94^, and Asp^95^ to alanine (3Ala) in VEGF-DΔNΔC was sufficient to prevent phosphorylation of VEGFR-3 (Fig. 4*B*, *right*). In contrast, for VEGF-CΔNΔC, alanine exchange of the three, five, six, seven, or eight residues N-terminal to Ser^121^ (3Ala, 5Ala, 6Ala, 7Ala, or 8Ala, respectively) did not have any pronounced effect on phosphorylation of either VEGFR-2 or VEGFR-3 (Fig. 4*C*). Likewise, a mutant of VEGF-CΔNΔC in which residues 113–121 had been deleted (designated Δ9) induced phosphorylation on tyrosine of both VEGFR-2 and VEGFR-3, as did two mutants lacking residues 113–115 or 113–118 (designated Δ3 and Δ6, respectively) (Fig. 4*D*). These three mutants also promoted binding and cross-linking of the extracellular domains of VEGFR-2 and VEGFR-3, as assessed in bioassays (Fig. 4*D*).

Our studies of receptor phosphorylation shown in Fig. 4 indicate that the N-terminal region of mature VEGF-D, from residue Phe^93^ to Thr^98^, is critical for the activation of VEGFR-3 but not VEGFR-2. Surprisingly, residues in the homologous region of VEGF-C (*i.e.* from His^113^ to Lys^120^) are not critical for the activation of either VEGFR-2 or VEGFR-3 by this ligand.

##### Key Residues for Driving Proliferation and Migration of LECs Are Distributed Differently in the N-terminal α-Helices of Mature VEGF-D and VEGF-C

**FIGURE 5.**
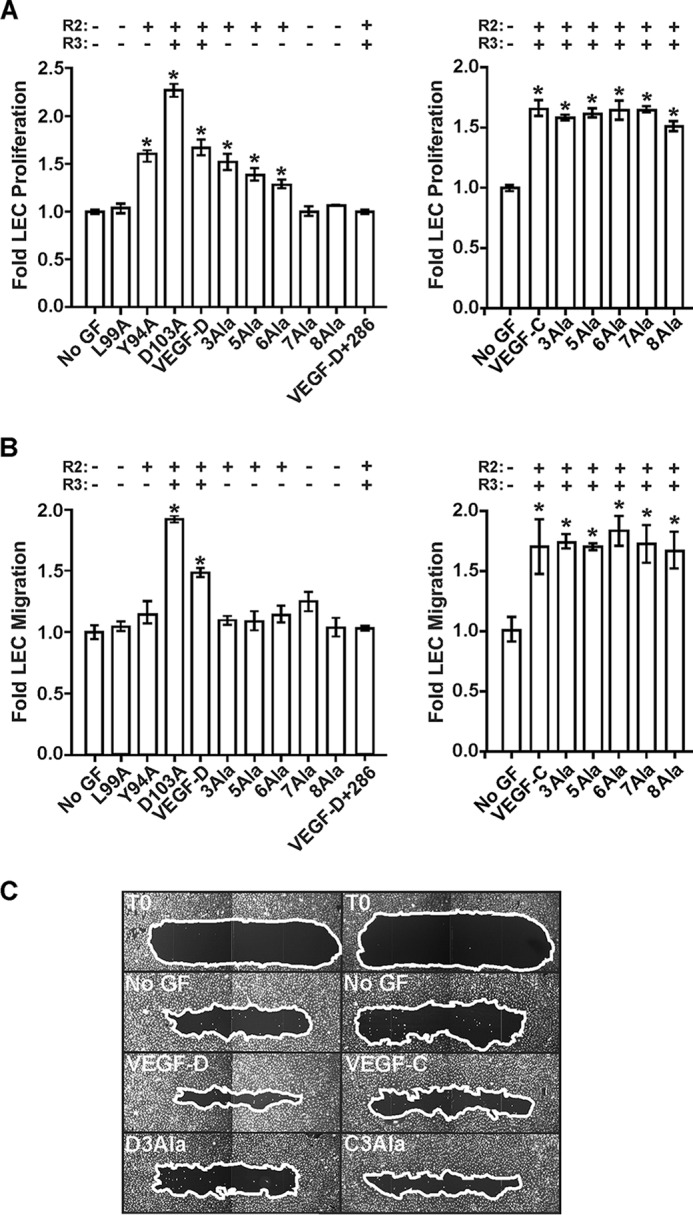
**Analyses of the role of N-terminal α-helices of mature VEGF-D and VEGF-C for proliferation and migration by LECs.** *A*, LEC proliferation assays. Adult LECs were treated with VEGF-DΔNΔC (*VEGF-D*), VEGF-CΔNΔC (*VEGF-C*), or their variants or left untreated (*No GF*). *VEGF-D*+*286*, combination of VEGF-DΔNΔC and a 10-fold molar excess of mAb 286. *y* axes represent proliferation by LECs stimulated with growth factor relative to that of unstimulated cells. *x* axes denote VEGF-D variants (*left*) and VEGF-C variants (*right*) used in assays. *B*, LEC migration assay. The capacity of variant proteins to induce cell migration was assessed in a scratch wound assay. Neonatal LECs were wounded, and the amount of wound closure was calculated for each variant as described under “Experimental Procedures.” *y* axes show migration of cells stimulated with growth factor relative to that of unstimulated cells. *x* axes denote VEGF-D variants (*left*) and VEGF-C variants (*right*) used in assays. *C*, images of selected scratch wounds. Wounds were imaged immediately post-wounding (*T0*, two examples) and after 24-h treatment with VEGF-DΔNΔC, VEGF-CΔNΔC, or the 3Ala variant of each (*D3Ala* and *C3Ala*, respectively). *No GF*, two results after 24 h with no growth factor. *White lines*, edges of the wounds. In *A* and *B*, the capacity of variants to activate VEGFR-2 (*R2*) or VEGFR-3 (*R3*) is indicated *above* the graphs, and *asterisks* indicate that results differ from *No GF* in a statistically significant fashion, as assessed by one-way analysis of variance with Tukey's post hoc test. The amounts of VEGF-D or VEGF-C variants were matched in each assay.

The variants of mature human VEGF-D described above provided the opportunity to assess the importance of residues in the N-terminal α-helix for the biological activities of this growth factor. Furthermore, given that these variants exhibited distinct receptor-binding specificities, they could also be used to assess the role of VEGFR-2 and VEGFR-3 in the bioactivities of VEGF-D. We focused on the proliferation and migration of LECs because these processes are required for the remodeling of lymphatics in cancer, which in turn promotes metastatic spread via the lymphatic vasculature (1). Migration of neonatal human dermal microvascular LECs was monitored in a scratch wound assay; see “Experimental Procedures” for details of the protocol. As expected, VEGF-DΔNΔC, which activates VEGFR-2 and VEGFR-3, promoted both proliferation and migration of LECs; these effects of VEGF-D were blocked by mAb 286 (Fig. 5, *A–C*). In contrast, VEGF-DΔNΔC variants Y94A, 3Ala, 5Ala, and 6Ala, all of which activate VEGFR-2 but not VEGFR-3, promoted proliferation but not migration of LECs (Fig. 5, *A–C*). Moreover, VEGF-DΔNΔC variants L99A, 7Ala, and 8Ala, which do not activate either VEGFR-2 or VEGFR-3, did not promote either proliferation or migration of LECs. VEGF-CΔNΔC, like VEGF-DΔNΔC, promoted both proliferation and migration of LECs in these assays, but in contrast to VEGF-D, the 3Ala, 5Ala, 6Ala, 7Ala, and 8Ala variants of VEGF-CΔNΔC (which activate both VEGFR-2 and VEGFR-3) all promoted proliferation and migration of LECs (Fig. 5, *A–C*). These data further confirm that the N-terminal region of mature VEGF-D, from residue Phe^93^ to Thr^98^, is a critical determinant of biological activity, whereas this is not the case for the homologous region of mature VEGF-C. Our findings also emphasize the importance of VEGFR-2 signaling for proliferation of LECs and of VEGFR-3 signaling for migration of these cells.

##### Enhancing the Capacity of VEGF-D to Activate VEGFR-3 Promotes Expression of COX-2 in LECs

**FIGURE 6.**
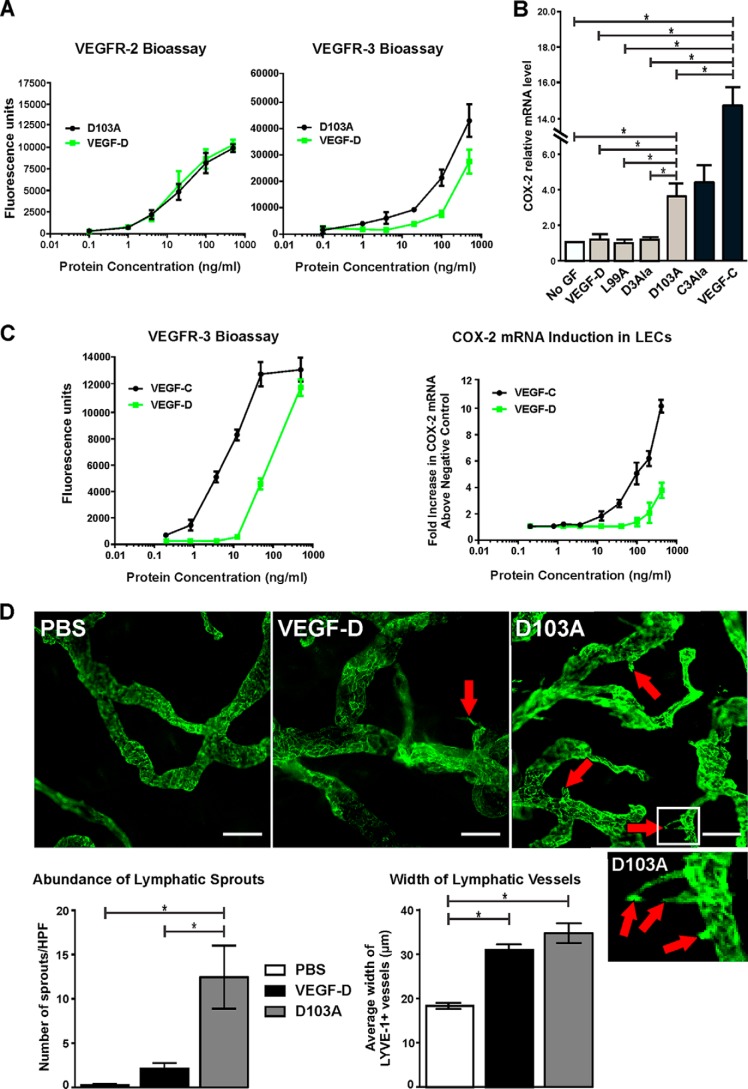
**Assessment of the D103A variant and VEGF-DΔNΔC for receptor interactions, stimulation of COX-2 expression, and sprouting lymphangiogenesis.** *A*, bioassays for binding and cross-linking of extracellular domains of VEGFR-2 (*left*) and VEGFR-3 (*right*) with VEGF-DΔNΔC (*VEGF-D*) and the D103A variant of VEGF-DΔNΔC. *Data points*, mean; *error bars*, S.D. *B*, effect of VEGF-DΔNΔC, the D103A variant, and other selected variants of VEGF-DΔNΔC (*gray bars*) and VEGF-CΔNΔC (*VEGF-C*) and the 3Ala variant of VEGF-C (*C3Ala*) (*black bars*) on the level of COX-2 mRNA in adult LECs as assessed by quantitative RT-PCR (*D3Ala* denotes the 3Ala variant of VEGF-DΔNΔC). Cells were exposed to 100 ng/ml ligands for 30 min before lysis for RNA preparation, as described under “Experimental Procedures.” COX-2 mRNA levels were normalized to β-actin and are expressed relative to the level in cells that were not treated with ligand (*No GF*). *Columns*, mean; *error bars*, S.D. *C*, titrations of VEGF-DΔNΔC and VEGF-CΔNΔC in the VEGFR-3 bioassay (*left*) and for the capacity to increase COX-2 mRNA levels in LECs (*right*). -Fold increases of COX-2 mRNA are relative to cells that were not treated with growth factor. In both graphs, *data points* indicate the mean, and *error bars* denote S.D. *D*, VEGF-DΔNΔC and the D103A variant (1 μg) were subcutaneously injected into ears of mice every 24 h for 3 days, as described under “Experimental Procedures”; PBS was the negative control. Ears were harvested and stained for lymphatics using antibody to LYVE-1 (*green*); the vessels shown are predominantly initial lymphatics. A high power image of the region within the *white rectangle* in the D103A image, showing three lymphatic sprouts, is shown *below* the lower power D103A image. *Red arrows* indicate lymphatic sprouts, which are quantitated in the *left-hand graph*; *scale bars*, 50 μm. *HPF*, high powered field. The width of LYVE-1-positive lymphatics is quantified in the *right-hand graph*. In both graphs, *columns* show mean and *error bars* denote S.E. In *B* and *D*, *asterisks* indicate statistically significant differences, as assessed by one-way analysis of variance with Tukey's post hoc test.

The VEGF-D variant D103A exhibited stronger activity than VEGF-DΔNΔC in assays of binding, cross-linking, and tyrosine phosphorylation of VEGFR-3 (Fig. 2, *B–D*). To explore this further, we titrated D103A and VEGF-DΔNΔC in the bioassays of VEGFR-2 and VEGFR-3 binding and cross-linking. This analysis confirmed that D103A was more potent in the VEGFR-3 bioassay than VEGF-DΔNΔC, whereas these two proteins exhibited comparable potency in the VEGFR-2 bioassay (Fig. 6*A*). The data in Figs. 2 (*B–D*) and 6*A* show that the D103A mutant of VEGF-DΔNΔC allows assessment of the functional consequences of specifically enhancing the capacity of mature VEGF-D to activate VEGFR-3.

It was previously shown in an animal model of cancer that tumor-derived VEGF-C promoted expression of COX-2 in the endothelial cells of collecting lymphatic vessels, whereas VEGF-D did not, indicating distinct molecular mechanisms by which these two growth factors promote metastasis via lymphatics (5). Likewise, when AdLECs were treated *in vitro* with 100 ng/ml VEGF-CΔNΔC and VEGF-DΔNΔC, only the former induced higher levels of mRNA for COX-2, as assessed by quantitative RT-PCR (Fig. 6*B*). Treatment with the 3Ala variant of VEGF-CΔNΔC, which activates VEGFR-2 and VEGFR-3, also increased levels of COX-2 mRNA in AdLECs. In contrast, the L99A variant of VEGF-DΔNΔC, which exhibits decreased binding to both VEGFR-2 and VEGFR-3, did not alter the level of COX-2 mRNA; nor did treatment with the 3Ala variant of VEGF-DΔNΔC, which activates VEGFR-2 but not VEGFR-3. Unexpectedly, treatment of LECs with the D103A variant of VEGF-DΔNΔC was able to induce increased levels of COX-2 mRNA, in contrast to VEGF-DΔNΔC (Fig. 6*B*). This indicates that enhancing the potency of VEGF-D for VEGFR-3 activation promotes the capacity of this growth factor to drive increased expression of COX-2 by LECs.

To explore why VEGF-CΔNΔC at 100 ng/ml promoted COX-2 expression in LECs but VEGF-DΔNΔC did not, we conducted titrations of these ligands in this assay and in the bioassay of VEGFR-3 binding and cross-linking. This showed that VEGF-CΔNΔC is ∼10-fold more potent than VEGF-DΔNΔC for binding and cross-linking VEGFR-3 and for inducing COX-2 expression (Fig. 6*C*). These data demonstrate that VEGF-DΔNΔC can induce increased expression of COX-2 in LECs, but only at higher ligand concentrations than for VEGF-CΔNΔC. Overall, these findings show that the potency of VEGF family ligands for activating VEGFR-3 correlates with the capacity to promote expression of COX-2 in LECs.

##### Enhancing the Capacity of VEGF-D to Activate VEGFR-3 Promotes Lymphatic Sprouting

We assessed the D103A variant of VEGF-DΔNΔC in a model of sprouting lymphangiogenesis in the ears of mice to monitor *in vivo* effects of specifically enhancing the capacity of VEGF-D to activate VEGFR-3. This model involves delivery of VEGF-D to initial lymphatics in the dermis of adult skin via intradermal injection in the presence of Matrigel (see “Experimental Procedures”). Treatment with VEGF-DΔNΔC led to lymphatic vessels with more sprouts and a larger mean width than the PBS-negative control (Fig. 6*D*); the increase in mean vessel width was statistically significant. Notably, the D103A variant induced a very large number of sprouts on lymphatics, statistically significantly more than those induced by VEGF-DΔNΔC. However, the mean width of lymphatics in ears treated with D103A was comparable with those treated with VEGF-DΔNΔC. These data indicate that the D103A mutant is advantageous for promoting lymphangiogenic sprouting *in vivo*. This finding is consistent with our data showing that this variant drives enhanced migration and proliferation of LECs *in vitro*, compared with VEGF-DΔNΔC (see Fig. 5, *A* and *B*); both of these processes would be required for lymphatic sprouting based on analogy to angiogenic sprouting (40). These findings suggest that the D103A variant of VEGF-DΔNΔC may be useful for promoting therapeutic lymphangiogenesis designed to enhance lymphatic function in disease settings.

## Discussion

This study explores the molecular basis underlying functional differences between VEGF-C and VEGF-D. The starting point was to better define the interaction of VEGF-D with its receptors, given that, in contrast to VEGF-C, there have been no reports of structures for VEGF-D in complex with either VEGFR-2 or VEGFR-3. We used the neutralizing VEGF-D mAb 286 to identify a region of this growth factor, in the N-terminal α-helix of the mature form, which is important for receptor binding. Some of the single alanine substitutions that we generated in the mAb 286 binding epitope prevented binding of VEGF-D to VEGFR-2 and VEGFR-3, indicating that mAb 286 targets a region required for receptor binding rather than acting via steric hindrance. We identified an amino acid residue in the α-helix, Tyr^94^, that is critical for activating VEGFR-3 but not VEGFR-2 and showed that residues Leu^99^, Ile^102^, Glu^105^, and Trp^106^ are important for binding both receptors. Surprisingly, the region of VEGF-C homologous to residues Phe^93^–Lys^100^ of VEGF-D (*i.e.* VEGF-C residues His^113^–Lys^120^) is not required for binding VEGFR-2 or VEGFR-3, nor for VEGF-C to drive proliferation or migration of LECs. This is supported by our observation that a mutant of mature VEGF-C, in which residues 113–121 were deleted, is able to activate VEGFR-2 and VEGFR-3. These findings show that an N-terminal portion of the α-helix in mature VEGF-D (Thr^92^–Thr^98^) is important for binding VEGFR-3 but not VEGFR-2, whereas the remainder of this helix (Leu^99^–Thr^109^) is important for binding both receptors. In contrast, the corresponding N-terminal portion of the α-helix in mature VEGF-C (His^113^–Ser^121^) is dispensable for binding either receptor. It has been shown that some residues (*e.g.* Asp^123^, Trp^126^, and Arg^127^) in the remainder of the α-helix of mature VEGF-C (Ile^122^–Thr^129^) are important for binding VEGFR-2 and/or VEGFR-3 (36, 37). These observations raise the possibility of post-translational regulatory mechanisms targeting the N-terminal portions of the α-helices that could exert distinct effects on the receptor-binding specificities and biological activities of VEGF-C and VEGF-D.

Cleavage of the C-terminal propeptide from the VHD of VEGF-D occurs after residue Arg^205^ (25). Two forms of mature VEGF-D can then be generated by two distinct cleavage events that remove the N-terminal propeptide, one giving rise to an N terminus at Phe^89^ (VEGF-D(89–205)) and the other at Lys^100^ (VEGF-D(100–205)) (25). Our results suggest that these two derivatives exhibit different receptor-binding specificities; VEGF-D(89–205) would activate both VEGFR-2 and VEGFR-3, whereas VEGF-D(100–205) would not activate VEGFR-3. Further, our data on the importance of Leu^99^ for the VEGFR-2 interaction suggest that VEGF-D(100–205) would exhibit reduced binding and activation of VEGFR-2 compared with VEGF-D(89–205). These predictions are broadly consistent with a previous study showing that a variant of VEGF-D(89–195), with a C117A mutation, could activate both VEGFR-2 and VEGFR-3 (32). In contrast, a C117A variant of VEGF-D(100–195) was barely able to bind and cross-link the VEGFR-3 extracellular domain in bioassays and exhibited much weaker potency for activating VEGFR-3 than the C117A variant of VEGF-D(89–195). The VEGF-D(100–195) variant exhibited lower potency for binding and cross-linking of the VEGFR-2 extracellular domain compared with the VEGF-D(89–195) variant, as expected based on our data, but was able to activate this receptor. The capacity of this VEGF-D(100–195) variant to activate VEGFR-2, in contrast to the L99A mutant reported here, may in part be due to the C117A mutation which can increase the bioactivity of VEGF-D (the comparable mutation in VEGF-C has similar effects) (41–44). Overall, it is clear that the choice of site at which the N-terminal propeptide is cleaved influences receptor-binding specificity of the resulting mature form of VEGF-D.

Proteolytic cleavage of VEGF-C to remove the N-terminal propeptide was previously reported to occur at two distinct sites immediately after residue 102 or 111 (29). These sites are some considerable distance N-terminal to residues in the α-helix of mature VEGF-C important for receptor binding (*e.g.* Asp^123^ and Arg^127^) (36, 37), so the choice between these sites is unlikely to alter the receptor-binding specificity of mature VEGF-C. However, it has recently been reported that incubation of VEGF-C *in vitro* with high concentrations of plasmin leads to cleavage of the N-terminal propeptide between residues 127 and 128, thus removing almost the entire N-terminal α-helix of mature VEGF-C and generating a protein incapable of activating VEGFR-3 (45). In contrast, more limited exposure to plasmin generated VEGF-C able to activate VEGFR-3 (28, 45), although the cleavage site involved has not been reported. Hence, it is possible that distinct sites could be used for cleavage of the N-terminal propeptide *in vivo*, leading to different receptor specificities for the resulting forms of mature VEGF-C. The locations of the cleavage sites in VEGF-C and VEGF-D utilized in cancer and other pathologies have not yet been systematically investigated.

The importance of VEGFR-3 signaling for sprouting lymphangiogenesis is supported by our findings that mutants of VEGF-DΔNΔC deficient for VEGFR-3 activation (but which could activate VEGFR-2) (*e.g.* Y94A, 3Ala, 5Ala, and 6Ala) were unable to promote migration of LECs in contrast to VEGF-DΔNΔC. Further, the D103A mutant, which has increased potency for binding and cross-linking VEGFR-3, had enhanced capacity to promote sprouting of lymphatics *in vivo* compared with VEGF-DΔNΔC. These findings are consistent with previous reports that mature VEGF-C, which binds both VEGFR-2 and VEGFR-3, can potently induce lymphatic sprouting and lymphangiogenesis and that VEGFR-3-specific variants of VEGF-C or VEGF-D also promote lymphangiogenesis (32, 46). The VEGF-DΔNΔC variants Y94A, 3Ala, 5Ala, and 6Ala were able to promote proliferation of LECs *in vitro* and enlargement of lymphatic vessels *in vivo*, which is consistent with the notion that VEGFR-2 signaling promotes lymphatic vessel enlargement, as proposed previously (47). Our data also suggest that VEGFR-3 activation is important for driving increased levels of COX-2 mRNA in LECs, which is relevant to tumor biology, given that COX-2 can be important for tumor-associated lymphangiogenesis, dilation of collecting lymphatic vessels, and metastatic spread (5, 48). Both VEGF-CΔNΔC and the D103A mutant of VEGF-DΔNΔC, which exhibit enhanced potency for activating VEGFR-3 compared with VEGF-DΔNΔC, also exhibited enhanced potency for inducing increased COX-2 expression in LECs compared with VEGF-DΔNΔC. This suggests that the potency of a VEGF ligand for activating VEGFR-3 is an important determinant of its potency for driving enhanced COX-2 expression in LECs. An alternative explanation for these findings is that VEGF-CΔNΔC and the D103A mutant of VEGF-DΔNΔC can engage co-receptors or other signaling molecules in LECs (2, 49) that facilitate up-regulation of COX-2 expression, whereas VEGF-DΔNΔC cannot or does so less effectively. The mechanistic role of COX-2 in tumor lymphangiogenesis and the potential involvement of this protein in lymphatic sprouting are important issues that require further investigation in *in vitro* and *in vivo* models of lymphatic remodeling.

Our data complement a previous study, employing an alternative neutralizing VEGF-D mAb, that identified a region of loop L2 of mature VEGF-D (Asn^147^–Leu^151^) as critical for binding both VEGFR-2 and VEGFR-3 (38). The importance of this region in loop L2 for receptor interactions as well as of the α-helix, as indicated here, is consistent with the crystal structure of VEGF-C in complex with regions of VEGFR-2 and VEGFR-3 (36, 37). In particular, the VEGF-C·VEGFR-2 complex allowed identification of an interface on VEGF-C, important for binding VEGFR-2, consisting of the N-terminal α-helix and the region of loop L2 from Asn^167^ to Leu^171^. This region of loop L2 in VEGF-C is homologous with residues Asn^147^–Leu^151^ in loop L2 of VEGF-D. Thus, the same region of loop L2 is important for both VEGF-C and VEGF-D to bind receptors. The α-helix is also critical, but our data show that the distribution of residues in the helix that are important for the VEGFR-3 interaction is different in VEGF-C and VEGF-D.

There is considerable interest in therapeutically targeting VEGF-C and/or VEGF-D in the clinic to block their action and thereby restrict angiogenesis, lymphangiogenesis, or vascular leakage in cancer, macular degeneration, and other conditions (9, 50–53). The VEGF-D mAb 286 characterized here, which blocks the binding and cross-linking of VEGFR-2 and VEGFR-3 by VEGF-D as well as the proliferation and migration of LECs induced by VEGF-D, could facilitate development of therapeutic monoclonal antibodies that block the action of VEGF-D or of bispecific antibodies that target both VEGF-D and VEGF-C. Such therapeutic antibodies could potentially be used in human cancer to restrict tumor angiogenesis, lymphangiogenesis, and lymphatic remodeling and thereby inhibit tumor growth and spread. Conversely, the delivery of VEGF family growth factors into tissues has the potential to promote therapeutic angiogenesis or lymphangiogenesis for treating cardiovascular conditions, lymphedema, and inflammatory diseases (9, 50, 54–60). Our finding that the D103A mutant of mature VEGF-D exhibits enhanced potency for VEGFR-3 could be of clinical significance because this protein, or derivatives thereof, could potentially be used therapeutically to drive lymphangiogenesis and lymphatic remodeling in lymphedema and inflammatory conditions. The aim of this approach would be to promote enhanced lymphatic function that has already been shown to be beneficial in clinically relevant animal models of these conditions (59, 61). Development of clinical agents designed to modulate the function of lymphatic vessels may have an impact in multiple prevalent human diseases and is a high priority for the future.

## Experimental Procedures

### 

#### 

##### Monoclonal Antibodies

mAb 286 was from R&D Systems (Minneapolis, MN), and VD1 (a neutralizing VEGF-D mAb) and VD4 (a mAb that binds, but does not neutralize, VEGF-D) have been described previously (39).

##### Protein Constructs

VEGF-DΔNΔC is a recombinant form of mature human VEGF-D that contains residues 93–201 of this growth factor and is N-terminally tagged with the FLAG octapeptide (22, 25). Likewise, VEGF-CΔNΔC is a form of mature VEGF-C containing residues 102–229 tagged with FLAG at the N terminus. Recombinant human VEGFR-2- and VEGFR-3-Fc chimeras (catalogue numbers 357-KD-050 and 349-F4–050, respectively) were from R&D Systems (Minneapolis, MN).

##### Site-directed Mutagenesis

Mutations of VEGF-C and VEGF-D were made in the regions ^113^HYNTEILKSIDNEWR^127^ and ^93^FYDIETLKVIDEEWQR^108^, respectively. Single mutations or mutations of multiple residues were introduced into constructs encoding FLAG-tagged or untagged VEGF-CΔNΔC or VEGF-DΔNΔC by amplification with specifically designed primers (see supplemental Table 1 for primers). All mutations were confirmed by nucleotide sequencing.

##### Protein Expression and Purification

Plasmids encoding VEGF-CΔNΔC, VEGF-DΔNΔC, or their variants were used for transient transfection of 293-F cells with the FreeStyle^TM^ MAX 293 expression system according to the manufacturer's instructions (Invitrogen). Cells expressing each variant were cultured in serum-free medium, and 30 ml of conditioned medium were collected 7 days post-transfection and used for analysis. Protein expression was tested by Western blotting with M2 anti-FLAG antibody or, for VEGF-D, with a mAb that targets the VHD (MAB2861, R&D Systems). Proteins were purified from conditioned medium by affinity chromatography on M2 (anti-FLAG) gel as described previously (25). Equal volumes of conditioned medium containing VEGF-DΔNΔC variants that were not tagged with the FLAG peptide were concentrated to the same final volume and buffer-exchanged into PBS using an Amicon size exclusion centrifugal filter with a 10 kDa nominal molecular mass limit (Millipore, Billerica, MA). The purity and concentrations of VEGF-C and VEGF-D variants were determined by Coomassie Brilliant Blue staining (see supplemental Fig. 1) and/or Western blotting compared with VEGF-C or VEGF-D standards of known concentration. Densitometry was performed using an Odyssey infrared imaging system (LI-COR Biosciences, Lincoln, NE).

##### Western Blotting

Variants of VEGF-DΔNΔC were resolved by SDS-PAGE, transferred to nitrocellulose membrane, probed with M2 anti-FLAG antibody (Sigma-Aldrich) or mAb 286 labeled with 800 IRDye® according to the manufacturer's instructions (LI-COR Biosciences), and detected with an Odyssey Infrared Imaging System. SDS-PAGE was carried out under reducing and denaturing conditions. Western blotting analysis to detect receptor phosphorylation was as described previously (62). For all Western blotting panels shown in the figures, each experiment was performed at least three times, and the same effects shown in the blots were observed each time the experiments were conducted.

##### ELISAs for Peptide Screening and Analyses of Ligand Binding by Antibodies and Receptors

For screening a synthetic biotinylated peptide library encompassing the VHD of VEGF-D (38), streptavidin high binding capacity coated plates (Reacti-Bind^TM^, Pierce) were incubated with 10 pmol of each peptide in PBS. Peptides were then blocked with 1% BSA in PBS containing 0.1% Tween 20 and incubated with 100 μl of either mAb 286 or M2 anti-FLAG antibody (2 μg/ml) for 1 h at room temperature. Bound mAb was detected with goat anti-mouse IgG coupled with horseradish peroxidase (HRP). Background was defined as signal detected in the absence of both antibody and peptide.

For analysis of binding of mAb 286 to VEGF-DΔNΔC variants, microtiter plates (Linbro®/Titertek®, ICN Biomedicals Inc., Aurora, OH) were coated with mAb 286 at 5 μg/ml in 100 mm carbonate buffer, pH 9.5, and then blocked with 1% BSA in PBS, 0.1% Tween 20 and incubated with 100 μl of serum-free cell culture medium containing 100 ng of VEGF-DΔNΔC variants for 1 h at room temperature. Bound VEGF-DΔNΔC was detected with an anti-VEGF-D antibody designated VD1 (39) coupled with HRP.

For testing receptor binding, microtiter plates were coated with human VEGFR-2- or VEGFR-3-Fc chimeras at 0.5 μg/ml in 100 mm carbonate buffer, pH 9.5, and then blocked with 1% BSA in PBS, 0.1% Tween 20 and incubated with 100 μl of PBS containing 20 ng of purified VEGF-C or VEGF-D variants for 1 h at room temperature. Bound ligands were detected with M2-HRP (Sigma-Aldrich) at 2 μg/ml for 1 h at room temperature. Assays were developed with an ABTS substrate system (Zymed Laboratories Inc., Carlsbad, CA) or with PrestoBlue^TM^ cell viability reagent (Invitrogen) and quantified by monitoring absorbance according to the manufacturers' instructions.

##### Bioassays for Binding and Cross-linking of Extracellular Domains of VEGFR-2 or VEGFR-3

Bioassays employed cell lines expressing chimeric receptors consisting of the entire extracellular domain of mouse VEGFR-2 or human VEGFR-3 and the trans-membrane and cytoplasmic domains of the mouse erythropoietin receptor (25, 63). Binding and cross-linking of the chimeric receptors allows these cells to survive and proliferate in the absence of IL-3. Bioassays with VEGF-C and VEGF-D variants were conducted as described previously (25, 64) except that the ligand concentration was 200 ng/ml (unless specified otherwise), and DNA synthesis or proliferation of cells was monitored using [^3^H]thymidine (65), a ViaLight Plus kit (Lonza, Basel, Switzerland), or Presto Blue^TM^ cell viability reagent (Invitrogen) according to the manufacturers' protocols. For some assays, mAbs VD1 and VD4 were included as controls.

##### Receptor Phosphorylation Assays

Phosphorylation of VEGFR-2 and VEGFR-3 on adult LECs (AdLECs, Lonza) treated with VEGF-C or VEGF-D variants at 200 ng/ml was analyzed as described previously (62).

##### Quantitative RT-PCR to Analyze COX-2 mRNA

AdLECs were serum-starved overnight and then exposed to VEGF-C or VEGF-D variants (at 100 ng/ml, unless stated otherwise) before isolation of total RNA using an RNeasy minikit (Qiagen, Valencia, CA) and preparation of cDNA with a high capacity cDNA reverse transcription kit (Thermo Fisher Scientific, Waltham, MA) using 1 μg of total RNA. Quantitation of cDNA for COX-2 and the internal reference gene (β-actin) was carried out with TaqMan Fast Universal PCR Master Mix using an Applied Biosystems 7500 fast real-time PCR machine (both from Thermo Fisher Scientific). TaqMan gene expression assays for COX-2 (HS00153133-M1) and β-actin (HS99999903-M1) were from Applied Biosystems (Thermo Fisher Scientific). Each reaction was done in triplicate, and all samples were analyzed using StepOne^TM^ Software version 2.2 (Thermo Fisher Scientific). Quantitation of COX-2 mRNA after treatment of cells with growth factors, relative to untreated control cells, was determined by the Δ*C_T_* method. Data are presented as mean ± S.D. of three independent experiments.

##### Cell Migration Assay

The migration of neonatal human dermal lymphatic microvascular endothelial cells (Clonetics, HMVEC-dLyNeo, Lonza) was assessed in a scratch wound assay. Cells were cultured in EGM^TM^-2MV growth medium (Lonza) with 5% FBS and supplements according to the manufacturer's instructions in humidified 5% CO_2_ at 37 °C. Cells (1 × 10^4^) were seeded in 96-well clear bottom imaging plates (Greiner Bio One, Frickenhausen, Germany) coated with 5 μg/ml fibronectin and grown to confluence. Before scratch wounding of the monolayer, cells were stained with Celltracker^TM^ Green CMFDA (Invitrogen, Thermo Fisher Scientific) for 45 min at 37 °C, and a 96-pin wounding device (V&P Scientific, San Diego, CA) was used to create a uniform scratch (∼0.5 × 5 mm). Immediately post-wounding, variants of VEGF-C and VEGF-D (200 ng/ml) in EBM-2 basal medium (Lonza) supplemented with 2% FBS were added to the cells, and each well was imaged using a BD Pathway 435 high throughput bioimager (BD Biosciences). The entire wound was captured using a 2 × 1 montage with a Nikon ×4 objective. After 24 h, cells were fixed with 4% paraformaldehyde (ProSciTech, Thuringowa, Queensland, Australia), blocked, and permeabilized in PBS containing 0.2% Triton X-100 and 2% BSA, stained with Phalloidin Alexa 488 (Invitrogen, Thermo Fisher Scientific), and imaged as above. Captured images were exported to Metamorph® (Molecular Devices, Sunnyvale, CA) or to FIJI (66) image processing software packages for analysis of wound closure using custom-designed macros.

##### Cell Proliferation Assays

AdLECs were grown to 90% confluence and starved overnight in EGM^TM^-2MV growth medium (Lonza) containing 2% FBS, 50 μg/ml gentamicin, and 2.5 μg/ml amphotericin B. Cells were trypsinized and counted, and ∼1.5 × 10^4^ cells were resuspended in 100 μl of medium containing VEGF-C or VEGF-D variants at 200 ng/ml. Cells were plated on wells of a clear bottom 96-well microplate (BD Biosciences) that had been coated with 5 μg/ml fibronectin and then incubated for 4 days. Cells were replenished at the 2-day time point with medium containing VEGF-C or VEGF-D variants. Cell proliferation was determined using CellTiter96®AQ_ueous_ One Solution cell proliferation assay reagent (Promega, Madison, WI) as described by the manufacturer.

##### Delivery of VEGF-D Variants in Vivo

Dermal delivery of purified VEGF-D variants in Matrigel plugs was performed essentially as documented (67) except that purified VEGF-D variants (1 μg) in 20 μl of PBS were mixed with 30 μl of Matrigel before injection (*i.e.* the variants were at 20 μg/ml in the injection solution). VEGF-D variants were injected subcutaneously every 24 h for 3 days.

##### Mice

SCID/NOD mice (8-week-old females) were from the Australian Resource Centre (Perth, Australia). Experiments were conducted according to ethical guidelines of the National Health and Medical Research Council of Australia and the Animal Ethics Committee of the Peter MacCallum Cancer Centre.

##### Structural Prediction of the mAb 286 Binding Site in the N-terminal α-Helix of Mature VEGF-D

The structure of the N-terminal α-helix (^93^FYDIETLKVIDEEWQ^107^) in human mature VEGF-D (presented in Fig. 2*A*), including the mAb 286 binding site, was generated from available crystallographic data for VEGF-D (Protein Data Bank code 2XV7) (32), with addition and optimization of missing side chains, using PyMOL (PyMOL Molecular Graphics System, version 1.3.x, Schrödinger, LLC, New York).

##### Statistical Analysis

All statistical comparisons were based on one-way analysis of variance using Tukey's post hoc test with significance level (α) at 0.05. Statistical analyses were performed with GraphPad Prism version 6.07 (GraphPad Software, La Jolla, CA).

## Author Contributions

N. D., N. C. H., M. I., S. R., and S. P.-F. conducted most of the experiments. N. D. and M. G. A. conceived the idea for the project. N. D., N. C. H., and M. G. A. wrote most of the paper. N. D., N. C. H., M. G. A., V. A. S., T. K., S. P.-F., S. P. W., and S. A. S. contributed to interpretation of the data and provided important intellectual content.

## Supplementary Material

Supplemental Data

## Footnotes

3 The abbreviations used are:

LEC: lymphatic endothelial cell
AdLEC: adult LEC
VEGFR: VEGF receptor
VHD: VEGF homology domain.
