# Supplementary material for: Differential Receptor Binding and Regulatory Mechanisms for the Lymphangiogenic Growth Factors Vascular Endothelial Growth Factor (VEGF)-C and -D
Source: J Biol Chem. 2016 Nov 16;291(53):27265–78. doi: 10.1074/jbc.M116.736801 (PMC5207153; doi:10.1074/jbc.M116.736801)
Supplement: Supplemental Data [file supp_291_53_27265__index.html]

Differential receptor binding and regulatory mechanisms for the lymphangiogenic growth factors VEGF-C and VEGF-D — Differential Receptor Binding and Regulatory Mechanisms for the Lymphangiogenic Growth Factors Vascular Endothelial Growth Factor (VEGF)-C and -D — Distinct Receptor Binding and Function of VEGF-C and VEGF-D — Supplemental Data 

# Differential Receptor Binding and Regulatory Mechanisms for the Lymphangiogenic Growth Factors Vascular Endothelial Growth Factor (VEGF)-C and -D

## Supplemental Data

- Supplemental Information (.pdf, 451 KB) - This file contains the Supplemental Information that should be accessible to the Reader.
