# Supplementary material for: Differential Receptor Binding and Regulatory Mechanisms for the Lymphangiogenic Growth Factors Vascular Endothelial Growth Factor (VEGF)-C and -D
Source: J Biol Chem. 2016 Nov 16;291(53):27265–78. doi: 10.1074/jbc.M116.736801 (PMC5207153; doi:10.1074/jbc.M116.736801)
Supplement: Supplemental Data [file 10.1074_M116.736801_jbc.M116.736801-1.pdf]

SUPPLEMENTAL INFORMATION

Differential Receptor Binding and Regulatory Mechanisms for the Lymphangiogenic Growth Factors VEGF-C and VEGF-D

Natalia Davydova, Nicole C. Harris, Sally Roufail, Sophie Paquet-Fifield, Musarat Ishaq, Victor A. Streltsov, Steven P. Williams, Tara Karnezis, Steven A. Stacker and Marc G. Achen

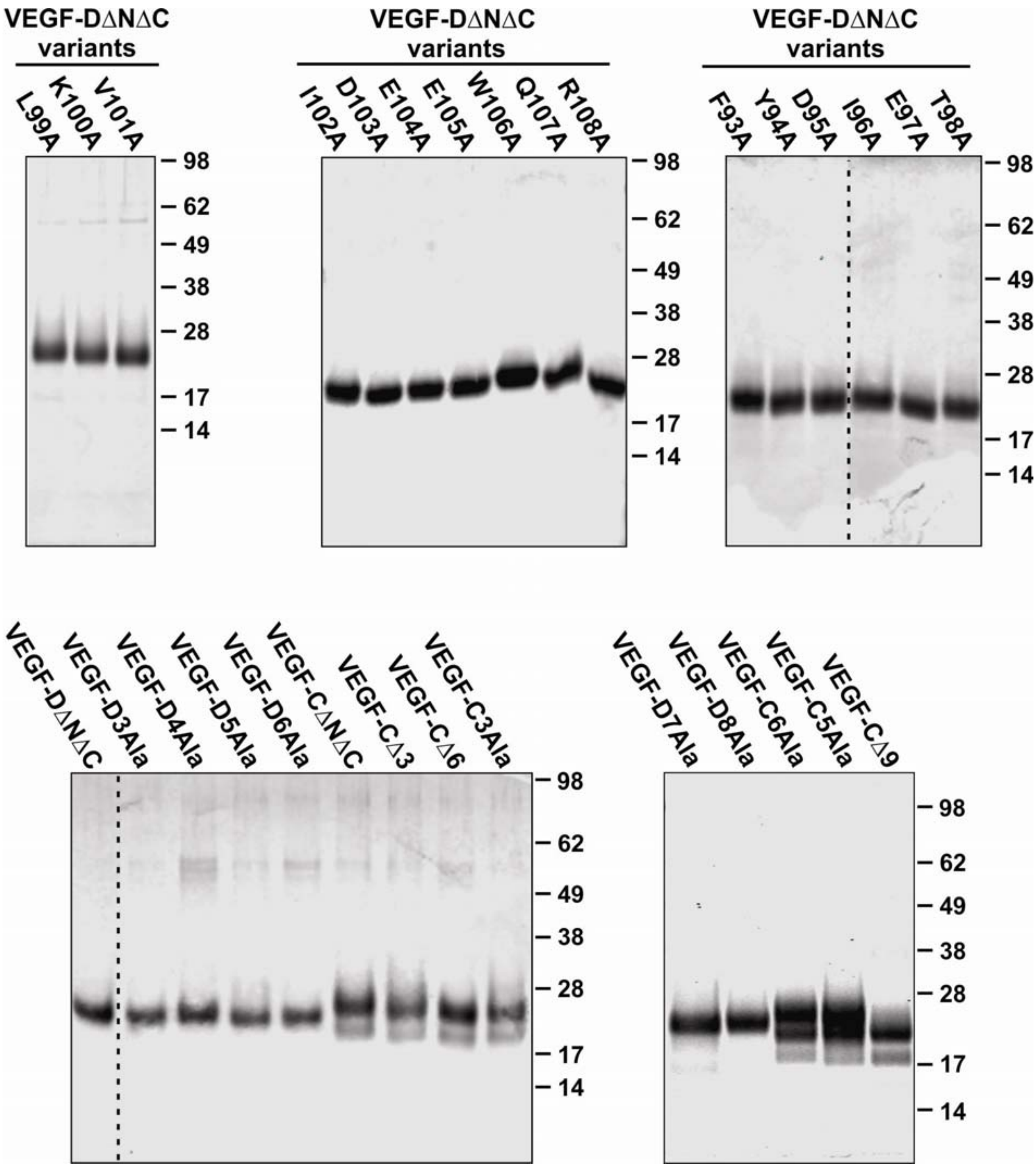

Supplementary Figure 1. Analysis of purified variants of VEGF-DΔNΔC and VEGF-CΔNΔC used in this study by SDS-PAGE and Coomassie staining. Proteins (approximately 1 μg) were subjected to SDS-PAGE under reducing conditions and stained with Coomassie Brilliant Blue. The expected sizes of the subunits of VEGF-DΔNΔC and VEGF-CΔNΔC variants are ~22 and ~24 kDa, respectively. The multiple bands detected in the 18 to 24 kDa range for variants of VEGF-CΔNΔC are likely due to variable degrees of glycosylation. Dotted lines indicate where irrelevant tracks have been removed from the images. Molecular weight markers in kDa are shown to the right of the images.

Supplementary Table 1. Nucleotide sequences of primers used to generate protein variants by site-directed mutagenesis.

Protein variant[1]	Primer pair sequence[2]
F93A	gacgacgacaagacgcgtaacgcctatgacattgaaacactaaa<br>tttagtgtttcaatgtcataggcgttacgcgtcttgtcgtcgtc
Y94A	gacgacgacaagacgcgtaatttcgctgacattgaaacactaaaagttat<br>ataacttttagtgtttcaatgtcagcgaaattacgcgtcttgtcgtcgtc
D95A	cgacaagacgcgtaacttctatgccattgaaacactaaaagttataga<br>tctataacttttagtgtttcaatggcatagaagttacgcgtcttgtcg
I96A	cgacaagacgcgtaacttctatgacgctgaaacactaaaagttatagatgaa<br>ttcatctataacttttagtgtttcagcgtcatagaagttacgcgtcttgtcg
E97A	gacgcgtaacttctatgacattgcaacactaaaagttatagatgaag<br>cttcatctataacttttagtgttgcaatgtcatagaagttacgcgtc
T98A	caagacgcgtaacttctatgacattgaagcactaaaagttatagatg<br>catctataacttttagtgcttcaatgtcatagaagttacgcgtcttg
L99A	gcgtaacttctatgacattgaaacagcaaaagttatagatgaagaatggcaa<br>ttgccattcttcatctataacttttgctgtttcaatgtcatagaagttacgc
K100A	gcgtaatttctatgacattgaaacactagcagttatagatgaagaatggcaa<br>ttgccattcttcatctataactgctagtgtttcaatgtcatagaaattacgc
V101A	tctatgacattgaaacactaaaagctatagatgaagaatggcaaagaac<br>gttctttgccattcttcatctatagcttttagtgtttcaatgtcataga
I102A	cttctatgacattgaaacactaaaagttgcagatgaagaatggcaaagaactca<br>tgagttctttgccattcttcatctgcaacttttagtgtttcaatgtcatagaag
D103A	cattgaaacactaaaagttatagctgaagaatggcaaagaactcag<br>ctgagttctttgccattcttcagctataacttttagtgtttcaatg
E104A	gaaacactaaaagttatagatgcagaatggcaaagaactcagtgc<br>gcactgagttctttgccattctgcatctataacttttagtgtttc
E105A	cactaaaagttatagatgaagcatggcaaagaactcagtgcag<br>ctgcactgagttctttgccatgcttcatctataacttttagtg
W106A	attgaaacactaaaagttatagatgaagaagcgcaaagaactcagtgcagc<br>gctgcactgagttctttgcgcttcttcatctataacttttagtgtttcaat
Q107A	aaagttatagatgaagaatgggcaagaactcagtgcagccctag<br>ctagggctgcactgagttcttgcccattcttcatctataacttt
R108A	gttatagatgaagaatggcaagcaactcagtgcagccctagag<br>ctctagggctgcactgagttgcttgccattcttcatctataac
Y94A (U)	gcttcaatctcggcgcgtaatttcgctgacattgaaacactaaaagttat<br>ataacttttagtgtttcaatgtcagcgaaattacgcgccgagattgaagc
K100A (U)	cgcgtaatttctatgacattgaaacactagcagttatagatgaagaatggcaaa<br>tttgccattcttcatctataactgctagtgtttcaatgtcatagaaattacgcg
I102A (U)	atttctatgacattgaaacactaaaagttgcagatgaagaatggcaaagaactcag<br>ctgagttctttgccattcttcatctgcaacttttagtgtttcaatgtcatagaaat
VEGF-D3Ala	caaggacgacgacgacaagacgcgtaacgccgctgccattgaaacactaaaagttatagatgaag<br>cttcatctataacttttagtgtttcaatggcagcggcgttacgcgtcttgtcgtcgtcgtccttg
VEGF-D5Ala	caagacgcgtaatgccgctgccgctgcaacactaaaagttatagatgaag<br>ttcatctataacttttagtgttgcagcggcagcggcattacgcgtcttg
VEGF-D6Ala	gacaagacgcgtaatgccgctgccgctgcagcactaaaagttatagatgaagaatg<br>cattcttcatctataacttttagtgctgcagcggcagcggcattacgcgtcttgtc
VEGF-D7Ala	cgctgcagcagcaaaagttatagatgaagaatggc<br>gcagcggcattacgcgtc
VEGF-D8Ala	tgcagcagcagcagttatagatgaagaatggcaaagaac<br>gcggcagcggcattacgc
VEGF-C3Ala	tcaaggacagaagagactataaaatttgctgcagcagctgctgctacagagatcttgaaaagtattgataatgagtggag<br>ctccactcattatcaatacttttcaagatctctgtagcagcagctgctgcagcaaattttatagtctcttctgtccttga
VEGF-C5Ala	tcaatacttttcaagatcgctgcagcagcagctgctgcag<br>ctgcagcagctgctgctgcagcgatcttgaaaagtattga
VEGF-C6Ala	tgctgcagcggccttgaaaagtattg<br>gcagctgctgcagcaaat
VEGF-C7Ala	tgcagcggccgcgaaaagtattgataatgagtgg<br>gcagcagctgctgcagca
VEGF-C8Ala	tgcagcggccgcggcaagtattgataatgagtggagaaagac<br>gcagcagctgctgcagca
VEGF-CΔ3	acttttcaagatctctgtattatatgctgcagcaaattttatagtctc<br>gagactataaaatttgctgcagcatataatacagagatcttgaaaagt
VEGF-CΔ6	aatacttttcaagatctctgtatttgctgcagcaaattttatagtctc<br>gagactataaaatttgctgcagcaaatacagagatcttgaaaagtatt
VEGF-CΔ9	agagactataaaatttgctgcagcaacagagatcttgaaaagtattga<br>tcaatacttttcaagatctctgttgctgcagcaaattttatagtctct

[1]All proteins are tagged with the FLAG octapeptide except those indicated by “(U)” which are untagged

[2]For each primer pair, the first line is forward primer and the second is reverse primer
